# Evaluation of Deep Learning-Based Automated Detection of Primary Spine Tumors on MRI Using the Turing Test

**DOI:** 10.3389/fonc.2022.814667

**Published:** 2022-03-11

**Authors:** Hanqiang Ouyang, Fanyu Meng, Jianfang Liu, Xinhang Song, Yuan Li, Yuan Yuan, Chunjie Wang, Ning Lang, Shuai Tian, Meiyi Yao, Xiaoguang Liu, Huishu Yuan, Shuqiang Jiang, Liang Jiang

**Affiliations:** ^1^ Department of Orthopaedics, Peking University Third Hospital, Beijing, China; ^2^ Engineering Research Center of Bone and Joint Precision Medicine, Beijing, China; ^3^ Beijing Key Laboratory of Spinal Disease Research, Beijing, China; ^4^ Institute of Computing Technology, Chinese Academy of Sciences, Beijing, China; ^5^ University of Chinese Academy of Sciences, Beijing, China; ^6^ Department of Radiology, Peking University Third Hospital, Beijing, China

**Keywords:** spine tumor, Turing test, deep learning, MRI, primary tumor

## Abstract

**Background:**

Recently, the Turing test has been used to investigate whether machines have intelligence similar to humans. Our study aimed to assess the ability of an artificial intelligence (AI) system for spine tumor detection using the Turing test.

**Methods:**

Our retrospective study data included 12179 images from 321 patients for developing AI detection systems and 6635 images from 187 patients for the Turing test. We utilized a deep learning-based tumor detection system with Faster R-CNN architecture, which generates region proposals by Region Proposal Network in the first stage and corrects the position and the size of the bounding box of the lesion area in the second stage. Each choice question featured four bounding boxes enclosing an identical tumor. Three were detected by the proposed deep learning model, whereas the other was annotated by a doctor; the results were shown to six doctors as respondents. If the respondent did not correctly identify the image annotated by a human, his answer was considered a misclassification. If all misclassification rates were >30%, the respondents were considered unable to distinguish the AI-detected tumor from the human-annotated one, which indicated that the AI system passed the Turing test.

**Results:**

The average misclassification rates in the Turing test were 51.2% (95% CI: 45.7%–57.5%) in the axial view (maximum of 62%, minimum of 44%) and 44.5% (95% CI: 38.2%–51.8%) in the sagittal view (maximum of 59%, minimum of 36%). The misclassification rates of all six respondents were >30%; therefore, our AI system passed the Turing test.

**Conclusion:**

Our proposed intelligent spine tumor detection system has a similar detection ability to annotation doctors and may be an efficient tool to assist radiologists or orthopedists in primary spine tumor detection.

## Introduction

Magnetic resonance imaging (MRI) is commonly used to diagnose spine disorders (e.g., myelopathy, spine canal stenosis, and traumatic injury). Spine tumors may cause spine fractures, instability, neurological deficits, or even paralysis. However, they are rarely observed because of their low incidence. Thus, it is difficult for junior radiologists or orthopedists to accumulate diagnostic experience, and they may not be capable of detecting different spine tumors on MRI. Deep learning (DL)—a class of artificial intelligence (AI)—is now prevalent in computer vision tasks. For spine imaging, especially MRI, DL, and other AI systems are being applied as diagnostic imaging technologies (1–5). Hallinan et al. (6) used a DL model for automated detection of the central canal, lateral recess, and neural foraminal stenosis in lumbar spine MRI; Huang et al. (7) utilized a DL-based fully automated program for vertebrae and disc quantifications on lumbar spine MRI; Merali et al. (8) developed a DL model for the detection of cervical spinal cord compression in MRI scans, and Ito et al. (9) developed the DL-based automated detection of spinal schwannomas in MRI. However, evaluation measures for AI methods are lacking because conventional radiology assessment systems do not meet the requirements of DL models. Thus, in this study, we applied the Turing test, a classical evaluation method in AI, on primary spine tumor DL detection on MR images.

Alan Turing, a British mathematician and theoretical computer scientist, is widely regarded as the founding father of AI. Alan Turing’s paper in 1950 entitled *“Computing Machinery and Intelligence”* had considered the question ‘‘Can machines think?’’ (10). Subsequently, he replaced the question with a significantly more practical scenario, namely, the Turing imitation game. The game has now become widely known, particularly in the clinical domain, as the Turing test. The Turing test (11, 12) is proposed to assess if a machine can think like a human, which reframed his question as follows: Can a machine display intelligence *via* imitation? Although this proposal is complex, a common operation of the Turing test requires an interrogator to communicate electronically with a subject to judge whether the subject is a human or machine (13, 14). The machine performed well if the interrogator makes an incorrect identification as often as a correct one. When evaluating the automated detection ability of a DL model, the gold standard is a comparison with the manual annotation of the same images by radiologists or orthopedists. However, the use of manual spine tumor annotations as the gold standard has been questioned because annotations themselves are subjective. For example, when a patient’s spine tumor is annotated by two different doctors, their annotations will hardly denote the same exact square, thereby reflecting inter- and intra-observer variability. Thus, the first purpose of using the Turing test was to confirm whether the automated detection ability of our DL models could achieve a clinically applicable standard compared with that of manual annotations at a tertiary university hospital. To this end, we proposed a simple interface program with choice questions to assess automated detection versus manual annotation based on position, shape, and area overlap. We hypothesized that if a clinical respondent is unable to distinguish the different bounding boxes drawn by an automated detection system and those produced manually by a spine expert, then it is likely that this DL model will be considered adequate for clinical application, which may assist orthopedists to find the primary spine tumors efficiently in the future. The second aim of this study was to assess the accuracy rate, false-positive, and false-negative results of the manual annotations from radiologists and orthopedists. It’s important to note that in this study, we mostly focused on the primary tumors located in the skeletal spine structures, thus we did not collect the intradural or intramedullary nervous system tumors.

## Methods

### Patient and Image Acquisition

We reviewed consecutive spinal tumor patients histologically diagnosed with primary spine tumors at our hospital between January 2012 and December 2020. Although primary spine tumors are rarely observed because of their low incidence, Peking University Third Hospital (PUTH) is a famous spine center in North China, and we can collect enough primary spine tumors patients in this study. The MR images of intradural or intramedullary nervous system tumors and ones acquired from other hospitals were excluded. Our database contained 508 patients, 226 women and 282 men (mean age, 49.0 [range, 3–84] years), including 19532 MR images with tumors. We used 12179 images from 321 patients to develop AI detection systems and 6635 images from 187 patients as a test set. For the Turing test, 100 patients were randomly selected from 187 patients in the test set. Sagittal and axial images were selected as representatives for manual annotation and training for the automated detection model because they span a wider range of spine regions, crucial for training the DL models for automated detection. Thus, the remaining 718 coronal images in the database did not participate in the training and testing process.

Preoperative MRI scans were performed on Discovery MR750 3.0T or Optima MR360 1.5T (GE Healthcare; Piscataway, NJ, USA). Conventional MRI scanning sequences included axial T2-weighted imaging (T2WI), sagittal T2WI, coronal T2WI, T1-weighted imaging (T1WI), and fat-suppressed T2WI scans. For axial and sagittal reconstruction, the scans were performed with the following parameters: field of view = 320 mm × 320 mm; matrix = 94 × 94; flip angle = 90; slice thickness = 3.0 mm; slice spacing = 3.3 mm; FS-T2WI turbo spin echo, repetition time (TR) = 2500–4000 msec, and echo time (TE) = 50–120 msec; and T1WI, TR = 400–800 msec, and TE = 10–30 msec.

### Turing Test of Spine Tumors Detection

The study was approved by the PUTH Medical Science Research Ethics Committee review board, which waived the need for informed consent as this was a retrospective review of a previous prospective study.

**Figure 1:**
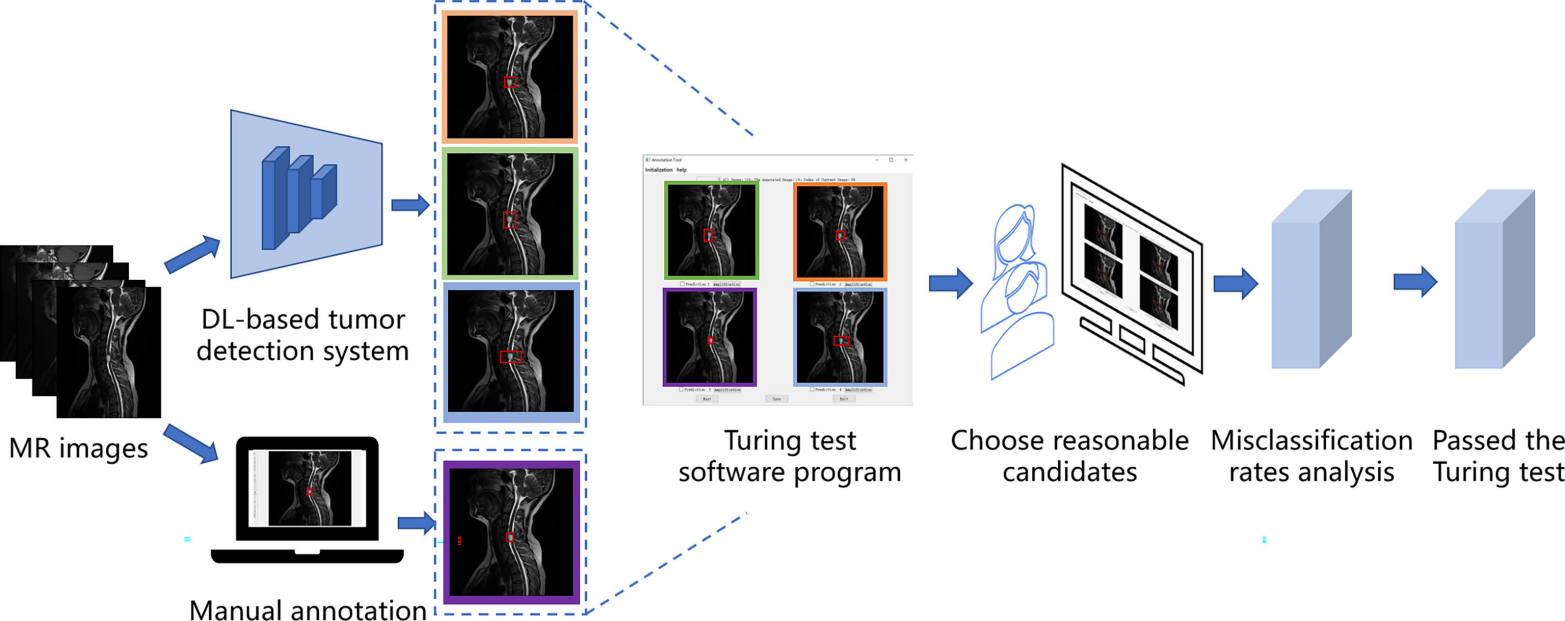
The flow of the Turing test. This figure shows the specific steps of the Turing test.

In our case, the Turing test was carried out with a choice question, each choice question featured four similar MR images as candidates, and three of the candidates were results predicted by DL models, one of them was annotated by doctors. Among them, the results predicted by the DL model were obtained by a DL-based tumor detection system with Faster Region-Convolutional Neural Network (Faster R-CNN) (15) architecture in our study, and only one was manually annotated by one of the five annotation doctors A-E (four radiologists and one orthopedist). One hundred patients and 200 choice questions (one axial choice question and one sagittal choice question for each patient), were randomly selected from our database for the Turing test. Without knowing which were human annotations, every choice question was shown to six respondent doctors F-K (four radiologists and two orthopedists) to select which one (reasonable candidate) among the four MR images was annotated by the annotation doctor. Since the DL-based tumor AI detection system is designed to react similarly to human intelligence, we considered the doctor’s lesion annotation as the correct option. Therefore, if the respondent did not correctly identify the image annotated by a human, his answer was considered a misclassification. The AI system passed the Turing test if the misclassification rates of the six respondents were all >30%. **Figure 1** shows the flow of the Turing test, which introduces the specific steps of the Turing test.


$$\text{Misclassification Rate}=\frac{F}{T+F}$$

where T represented the respondent correctly identifying the image annotated by a human, and F represented the respondent did not correctly identify the image annotated by a human.

### Manual Annotation Database

**Figure 2:**
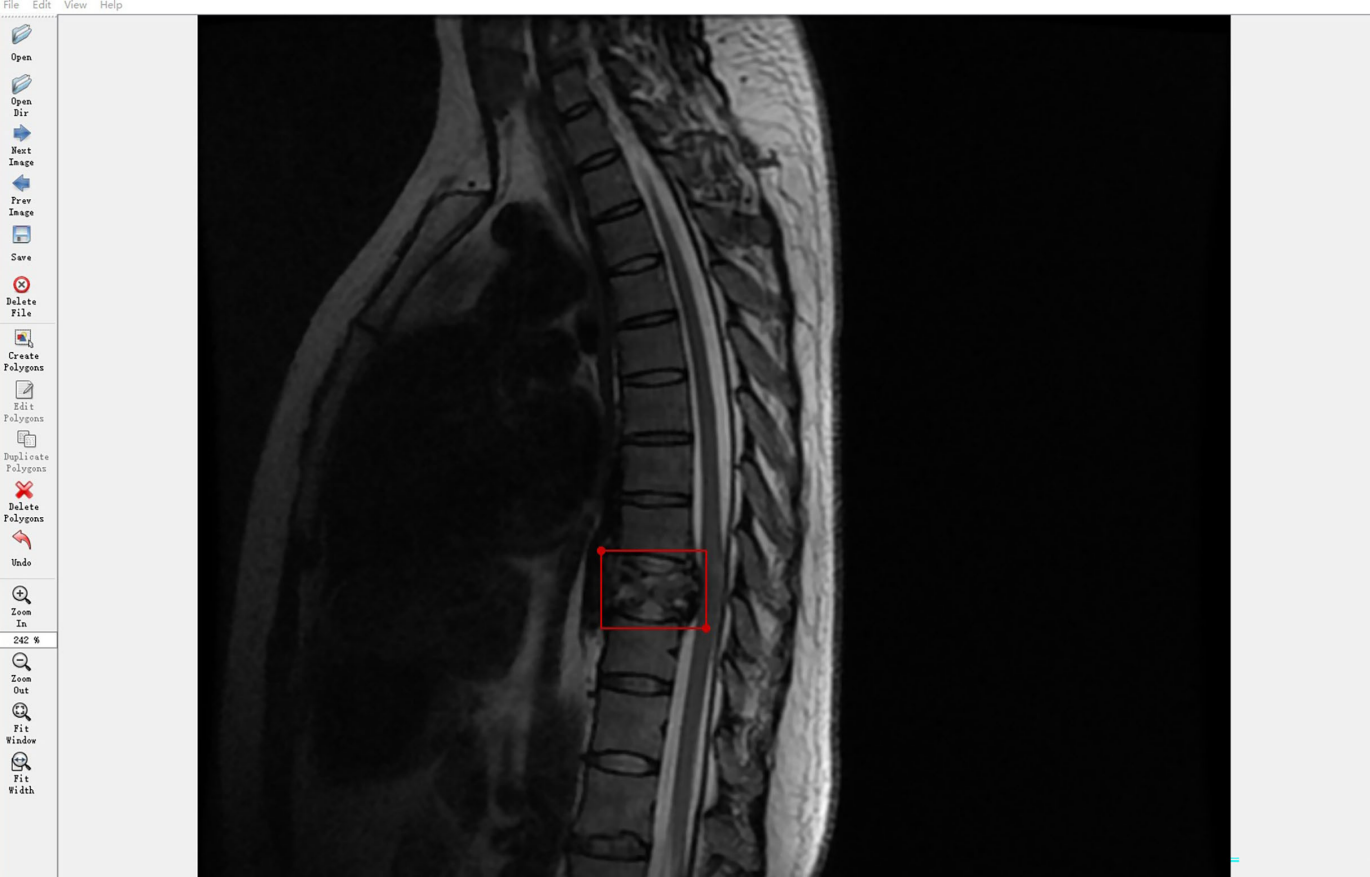
Labeling tool used by doctors to annotate tumor coordinates: Labelme. The Labelme displays the currently annotated image. The red annotation box indicates that the current location is a tumor. The annotation tool will automatically generate the coordinates of the upper left point and the lower right point.

Spine MRI data from Digital Imaging and Communications in Medicine files were exported in Joint Photographic Experts Group (JPEG) format from the picture archiving and communication systems of our hospital. These JPEG images were manually annotated using software *Labelme*, an image labeling tool developed in the Computer Science and Artificial Intelligence Laboratory at the Massachusetts Institute of Technology. *Labelme* is capable of creating customized labeling tasks or performing image labeling; we annotated the images by manually inputting a minimal bounding box containing every tumor lesion on each sagittal or axial MRI slice to generate JPEG images for the automated detection training (**Figure 2**). Taken together, four radiologists and one orthopedist (doctors A–E) annotated 19532 MRI slices. To ensure that each tumor was recognized by the DL model under different conditions, all slices on T1W1 and T2W1 MR images were annotated.

### Manual Annotation Assessment by Doctors

Before testing whether automated detection was sufficiently similar to manual annotation (namely, indistinguishable when judged by a blinded respondent), we randomly assessed the manual annotations to reduce inter- and intra-observer variability. The other three senior radiologists, except doctor F-K in our hospital, randomly and independently examined and verified the annotation images of doctors A–E. Based on the evaluation of the manual annotations, the computer engineers calculated the ultimate accuracy rate, false-positive rate, and false-negative rate of their labels by utilizing the confusion matrix. Clinical information of patients was not provided for any of the doctors to ensure a fair comparison between humans and DL models.

### Architecture of Deep Learning-Based Automated Detection

In this study, we trained the automated DL detection model using the locations and bounding box labels of spine tumors as training data. The automated detection model was trained and validated using a computer equipped with a Quadro P6000 graphics processing unit (NVIDIA; Santa Clara, CA), a Xeon E5-2667 v4 3.2 GHz CPU (Intel; Santa Clara, CA), and 64 GB of RAM.

We used PyTorch, a suitable framework for DL, to train a neural network model applied to the spine tumor dataset of MR images. A two-stage DL system with Faster R-CNN (15) architecture was used as the training model and consisted of a region proposal network (RPN) and region regression. The RPN was used to generate many anchors to get region proposals. It used SoftMax to recognize whether the anchors were positive or negative, the lesions are generally considered to be in positive anchors. Then the region regression could correct the positive anchors to obtain accurate proposals. Three different backbones of the proposed model were used to extract MR image feature maps, like ResNet-50 (16), ResNet-101 (16), and ResNet-152 (16), consisting of 50, 101, and 152 convolutional, pooling, and activation layers, respectively. These feature maps were shared for the RPN layer and region regression. And Feature Pyramid Networks (17) were also used in the model to solve the multi-scale problem in object detection.

**Figure 3:**
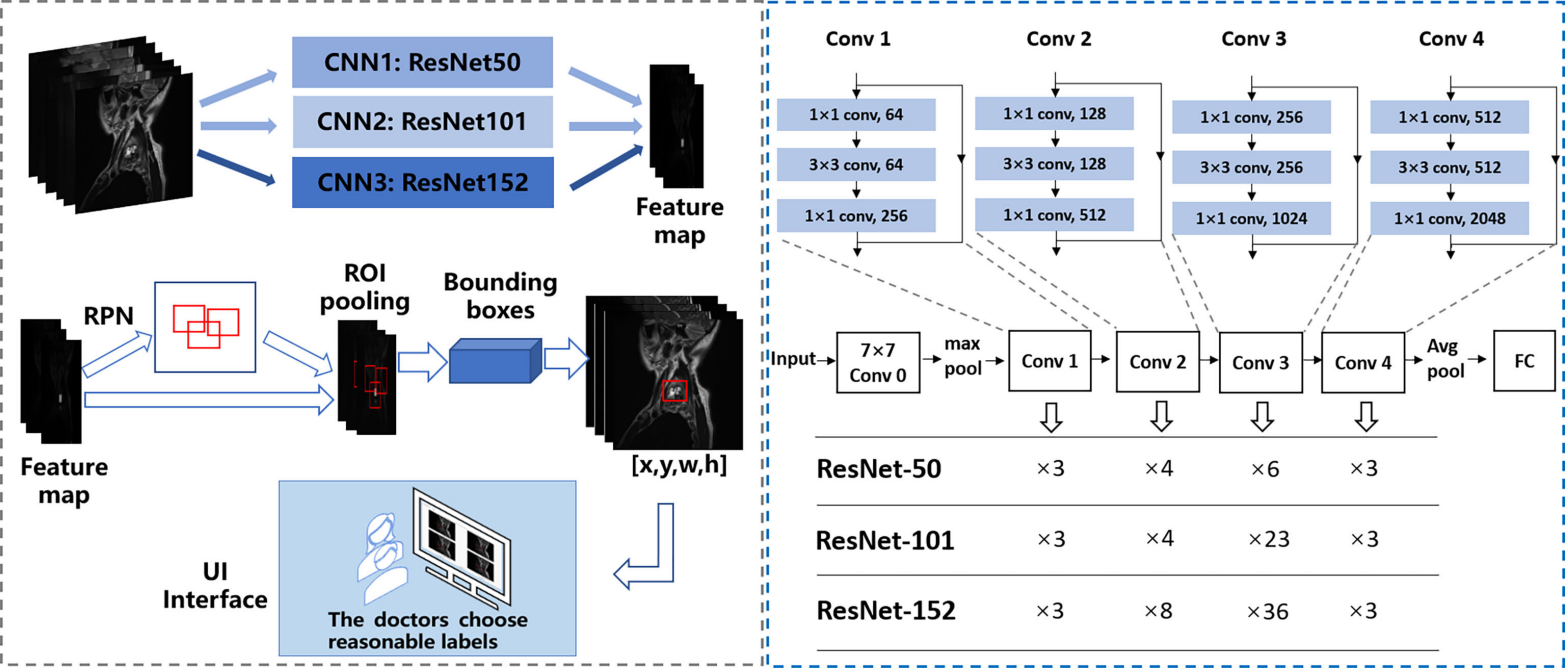
The framework of automated detection of spine tumors utilizing the Turing Test. Faster RCNN is used as the framework, and ResNet50, ResNet101, and ResNet152 are used to extract image features respectively.

The first-stage inputs were the MRI spine data of the three different backbones; the outputs were the different regions and activation maps, which were subsequently used as second-stage inputs. In the second stage, the region of interest (ROI) pooling layer collected the input feature maps and proposals, combining the information to extract proposal feature maps. Subsequently, a small network (i.e., multiple fully connected layers) was constructed with a regression branch to obtain the final precise positions of the lesion area. For efficient computing, all-region features were fed to the same regressor. Finally, we obtained the output of the three models. We call the Faster R-CNN framework with the backbone ResNet50, ResNet101, and ResNet152 as CNN1 (convolutional neural network 1), CNN2, and CNN3, respectively. **Figure 3** shows the automated detection framework of our Turing test.

### Evaluation Measurement in Artificial Intelligence

The tumor detection performance was evaluated from the aspect of the class label and position accuracy, which could be measured with average precision. Compared with the ground truth annotated by doctors, when the intersection of union (IoU) went over the threshold, the prediction was considered correct. The IoU formula is as follows:


$$IoU=\frac{b_{pred}{\;\cap }^{}\;b_{gt}}{b_{pred}\;\cup^{\text{ ​}}b_{gt}}$$

where, *b_pred_* and *b_gt_* represent a bounding box of predictions and ground truth, respectively.

### Training Implementation Details

In the training stage, the input images were divided into mini-batches. Each mini-batch contained eight images per GPU, and each image had 2000 region of interest samples with a ratio of one positive to three negatives. Specifically, anchors with an IoU >0.7 with the annotated bounding boxes) were set as positive examples; those with an IoU <0.3 were set as negative examples. The RPN anchors spanned five scales {16, 32, 64, 128, 256} and three aspect ratios {0.5, 0.8, 1.3}, totaling 15 anchors. The threshold of the non-maximum suppression layer was set to 0.5. We trained on one GPU with SGD for 10 epochs with a learning rate of 0.01, which was decreased by 0.5 every epoch. We used a weight decay of 0.0005 and momentum of 0.9. Due to the similarity between medical pictures, having more training pictures helps the DL model to better extract features, which can enhance the generalization of the model. Therefore, for better performance, axial and sagittal images were trained together for the MRI dataset. Similarly, T1W1 and T2W1 were trained together as a training set.

### Turing Test Software Program

To complete the Turing test, the annotated images were reviewed by a team of 6 respondents, including two radiologists and one orthopedist who worked at our hospital for approximately 10 years, and other two radiologists and one orthopedist who worked there >20 years. The six respondents (doctors F–K) specialized in spine tumors and had not performed the annotation previously (doctors A–E). The six respondents’ answering processes were double blindly designed to ensure no communication with any other people occurred.

**Figure 4:**
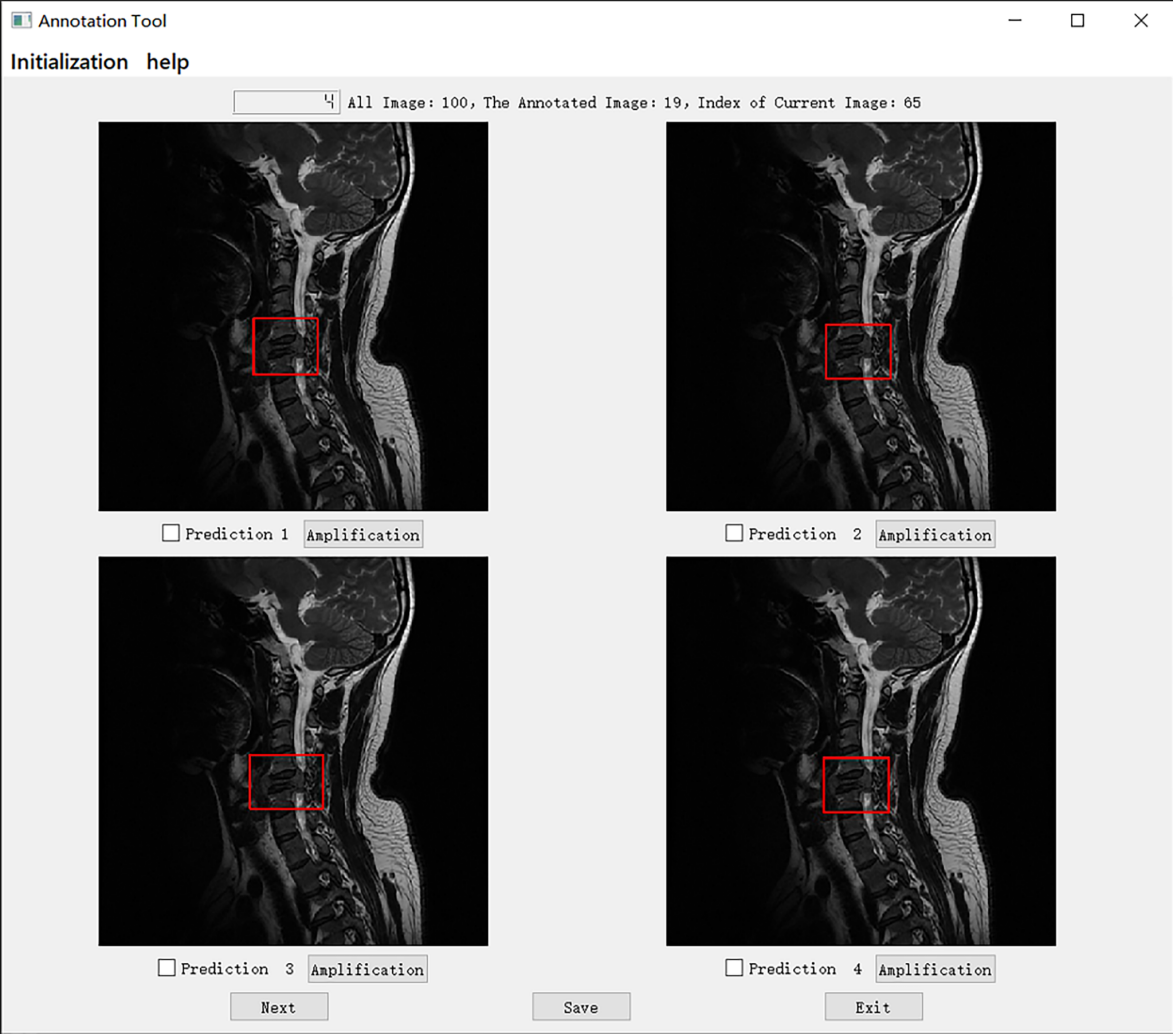
The choice interface of a Turing test software program. This software displays four options of one choice question, including amplification, timing, and technical functions. The user can click the next button to continue to the next question. After confirming the answer to the current question, click the save button to save the answer. After all the questions are completed, click the exit button to exit the program. Of these four options, prediction 3 is the result annotated by one of the doctors A-E, and prediction1, 2, and 4 is the result of the tumor location predicted by the model CNN 3, CNN1, and CNN2 respectively.

We set up a Turing test software program with choice questions (**Figure 4**). In every choice question, the respondents were shown an interface with four MR images of an identical tumor; three featured bounding boxes were generated by DL models, whereas only one featured a bounding box drawn by an annotation doctor. The four images with correspondent bounding boxes featured were randomly ordered in each question. The respondents would be asked, “Which one is annotated by a human?” Each respondent reviewed approximately 200 choice questions (sagittal and axial figures) from 100 patients, randomly selected from a pool of 6635 annotated images of the test set. Figures of the interfaces were presented for assessment in questions only once owing to the random nature of the selection process. The display could be adjusted to a standard window, and a magnifying tool was provided to enable a detailed image inspection. Additionally, the software program documented respondent responses provided for each question and the time required to choose each respondent.

Specifically, in the selection of questions shown in **Figure 4**, prediction 3 is the result annotated by one of the doctors A-E, and predictions 1, 2, and 4 is the tumor location predicted by models CNN 3, CNN1, and CNN2, respectively. The network depth of the models CNN 1, CNN 2, and CNN3 differed. Compared with CNN 1, CNN 2 and CNN3 have a sequentially increasing number of network layers; the more the layers of the network mean the richer the abstract features of different levels that can be extracted. Moreover, the deeper the network, the more abstract the features, and the more semantic information.

### Statistical and Data Analyses

All statistical analyses were performed using the Statistical Package for the Social Sciences (SPSS, version 26.0; IBM Corporation, Armonk, N.Y., USA). Results were obtained for the fivefold cross-validation of object detection. The Mann–Whitney U test and chi-squared test were used for comparisons between groups for continuous and categorical variables, respectively. A *P*-value <0.05 was considered significant. The criteria of true detection and false detection were calculated for the DL-based automated tumor detection on MR images and the annotation team.

## Results

### Patient Characteristics and Data Split

We obtained the MRI dataset of the primary spine tumors to train and evaluate our model. We trained together in these two views and tested them separately. Concerning primary spine tumors, 19532 images from 508 patients were included in the MRI dataset. We chose 12179 images from 321 patients to develop AI detection systems, including 7788 images (2346 axial; 5442 sagittal) randomly selected from 193 patients as the training set and 4391 images (1199 axial; 3192 sagittal) from 128 patients randomly selected as the validation set. The validation set was used to determine the network structure and help train a better model. Moreover, 6635 images (1835 axial; 4800 sagittal) from the other 187 patients were randomly selected as test set and Turing test data source. The dataset only contained axial and sagittal views, and the remaining 718 coronal images were not used for the training or the Turing test.

### Evaluation Measurement Among Doctors

**Figure 5:**
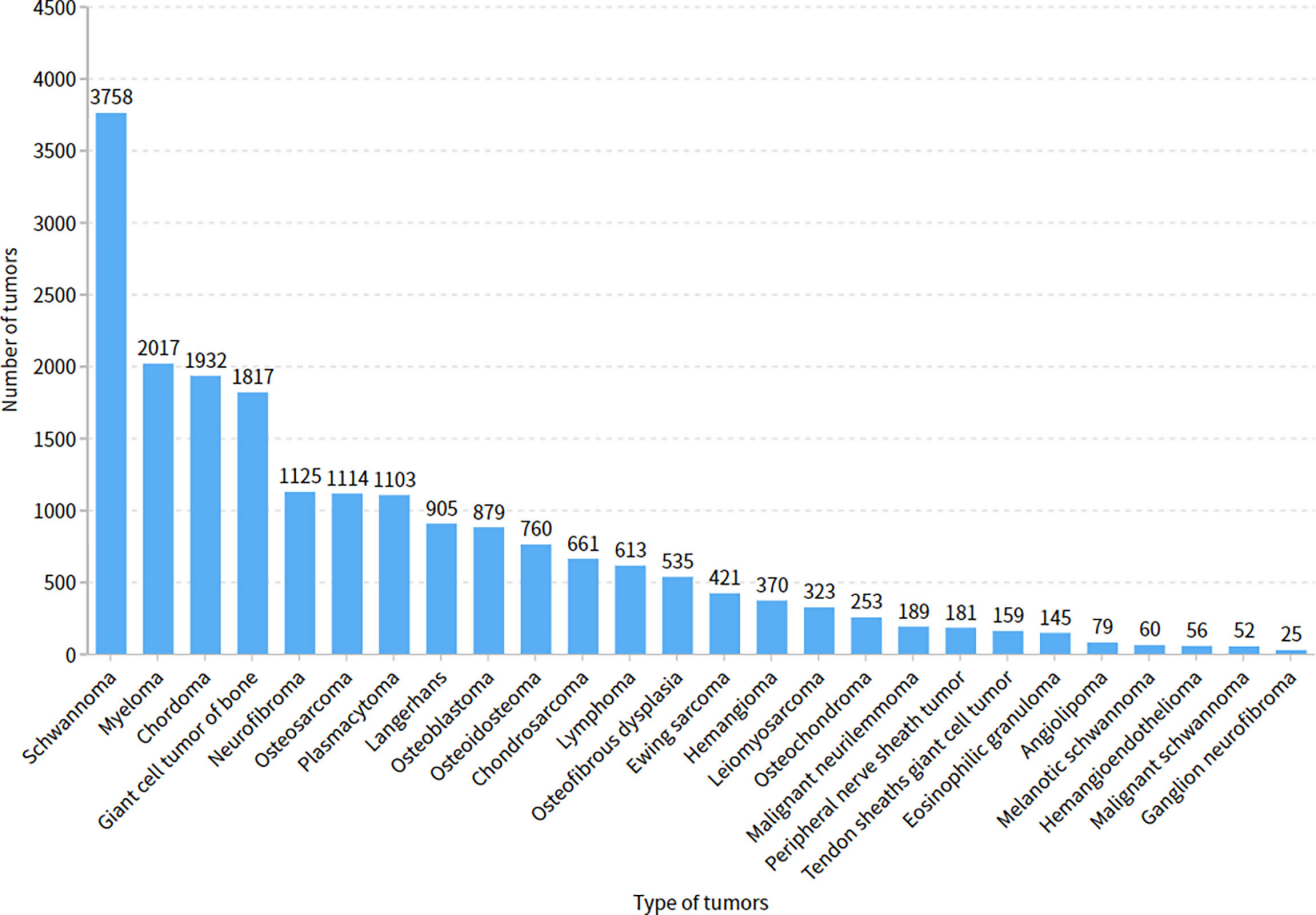
The number of images of different tumor categories.

**Figure 6:**
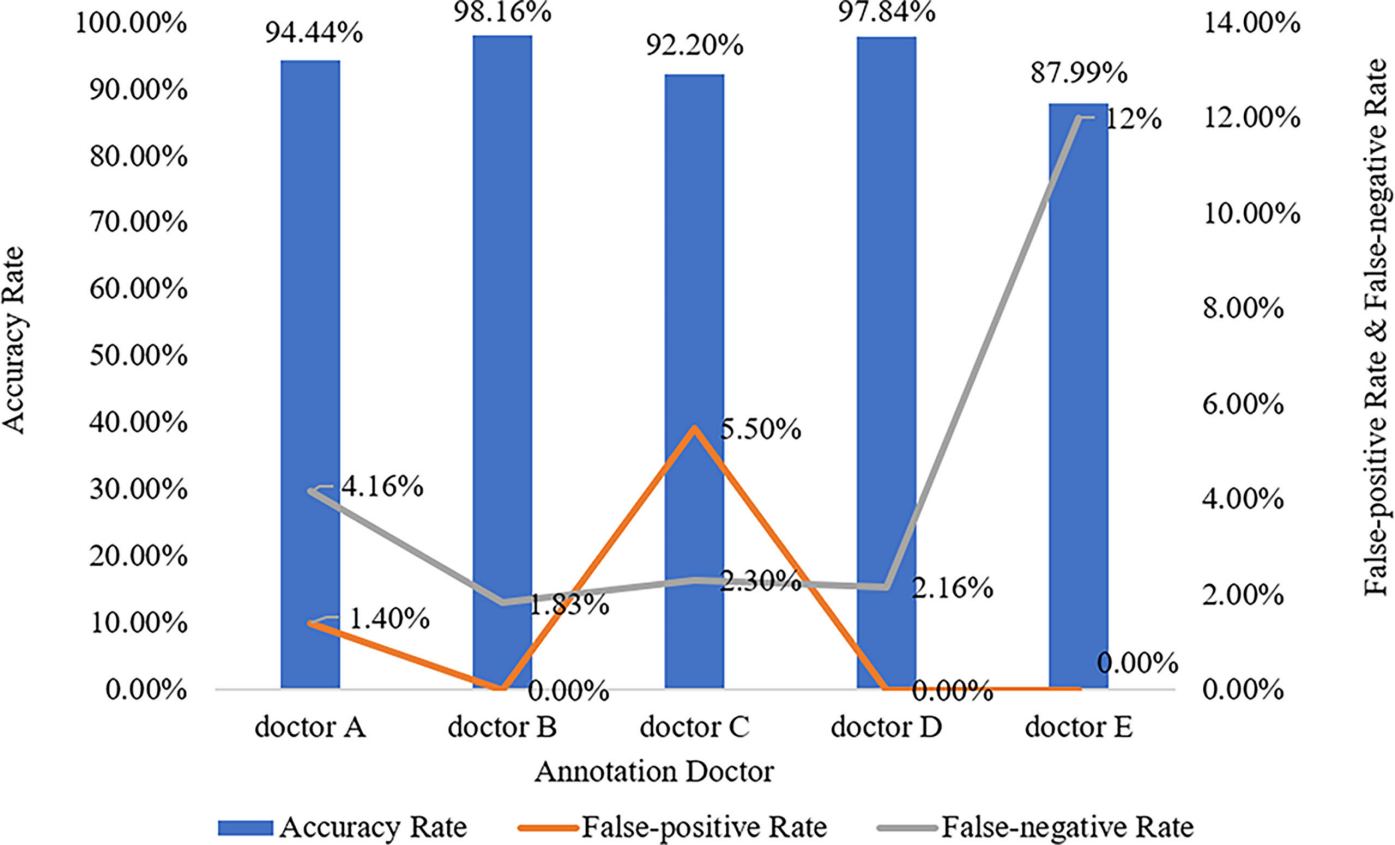
Manual annotation results on MRI primary spine tumor dataset in training set. This figure shows in detail the accuracy rate, false-positive rate, and false-negative rate of the training set annotated by doctors A-E.

**Figure 7:**
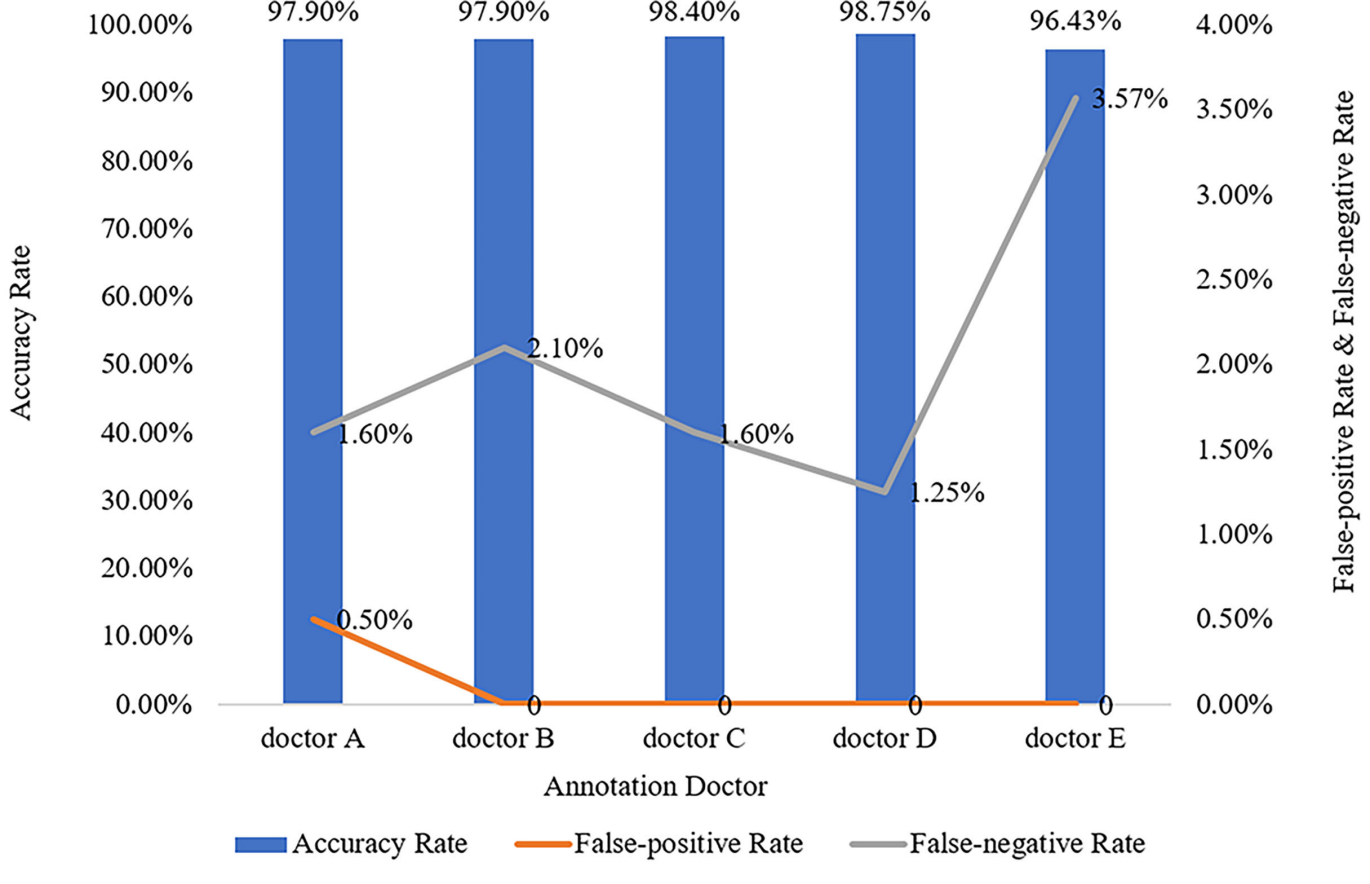
Manual annotation results on MRI primary spine tumor dataset in testing set. This figure shows in detail the accuracy rate, false-positive rate, and false-negative rate of the testing set annotated by doctors A-E.

The five doctors annotated primary spine tumors on MR images; the total number of annotated images for each doctor was 4527 (A), 4159 (B), 3910 (C), 3727 (D), and 3209 (E). As **Figure 5** shows, there were a total of 26 tumor histological categories in our dataset, such as schwannoma, myeloma, and chordoma, among others. In the dataset, there were 3758 schwannoma images and only 25 ganglion neurofibroma images. The evaluations of the five annotation doctors are listed in **Figures 6**, **7** and include detailed accuracy rate, false-positive rate, and false-negative rate of the spine tumors MRI manual annotations for each doctor. In the training set of primary spine tumors, the five doctors’ MRI annotations accuracy rates were 94.44% (A), 98.16% (B), 92.20% (C), 97.84% (D), and 87.99% (E); the false-positive rates were 1.40% (A), 0.00% (B), 5.50% (C), 0.00% (D), and 0.00% (E); the false-negative rates were 4.16% (A), 1.83% (B), 2.30% (C), 2.16% (D), and 12.00% (E). The average accuracy rate, false-positive rate, and false-negative rate of doctors A-E were 94.13%, 1.38%, and 4.49% respectively. In the test group of primary spine tumors, the five doctors’ MRI annotations accuracy rates were 97.90% (A), 97.90% (B), 98.40% (C), 98.75% (D), and 96.43% (E); the false-positive rates were 0.50% (A), 0.00% (B), 0.00% (C), 0.00% (D), and 0.00% (E); the false-negative rates were 1.60% (A), 2.10% (B), 1.60% (C), 1.25% (D), and 3.57% (E). The average accuracy rate, false-positive rate, and false-negative rate of doctors A-E were 97.88%, 0.10%, and 2.02% respectively. **Tables 1** and **2** show the details of the precision, recall, F1-score, specificity, and sensitivity in the training and testing sets.

**Table 1:** The details of precision, recall, f1 score, specificity, and sensitivity of the training set annotated by the doctor A-E.

	Doctor A	Doctor B	Doctor C	Doctor D	Doctor E
**Precision**	98.60%	100.00%	94.50%	100.00%	100.00%
**Recall**	95.95%	98.20%	97.62%	97.89%	89.29%
**F1 score**	97.26%	99.09%	96.04%	98.93%	94.34%
**Specificity**	98.56%	100.00%	94.67%	100.00%	100.00%
**Sensitivity**	95.95%	98.20%	97.62%	97.89%	89.29%

Precision = TP/(TP+FP); Recall = TP/(TP+FN); F1 score= (2*Precision*Recall)/(Precision + Recall);

Specificity = TN/(FP+TN); Sensitivity = TP/(TP+FN).

TP = true-positive: It is actually a lesion area, and the doctor annotated it as a lesion area;

FP = false-positive: It is actually not a lesion area, but the doctor annotated it as a lesion area;

FN = false-negative: It is actually a lesion area, but the doctor annotated it is not a lesion area;

TN = true-negative: It is actually not a lesion area, and the doctor annotated it is not a lesion area.

**Table 2:** The details of precision, recall, f1 score, specificity, and sensitivity of the testing set annotated by the doctor A-E.

	Doctor A	Doctor B	Doctor C	Doctor D	Doctor E
**Precision**	99.50%	100.00%	100.00%	100.00%	100.00%
**Recall**	98.42%	97.94%	98.43%	98.77%	96.55%
**F1 score**	98.96%	98.96%	99.21%	99.38%	98.25%
**Specificity**	99.48%	100.00%	100.00%	100.00%	100.00%
**Sensitivity**	98.42%	97.94%	98.43%	98.77%	96.55%

Precision = TP/(TP+FP); Recall = TP/(TP+FN); F1 score= (2*Precision*Recall)/(Precision + Recall);

Specificity = TN/(FP+TN); Sensitivity = TP/(TP+FN).

TP = true-positive: It is actually a lesion area, and the doctor annotated it as a lesion area;

FP = false-positive: It is actually not a lesion area, but the doctor annotated it as a lesion area;

FN = false-negative: It is actually a lesion area, but the doctor annotated it is not a lesion area;

TN = true-negative: It is actually not a lesion area, and the doctor annotated it is not a lesion area.

### Evaluation With the Turing Test

**Figure 8:**
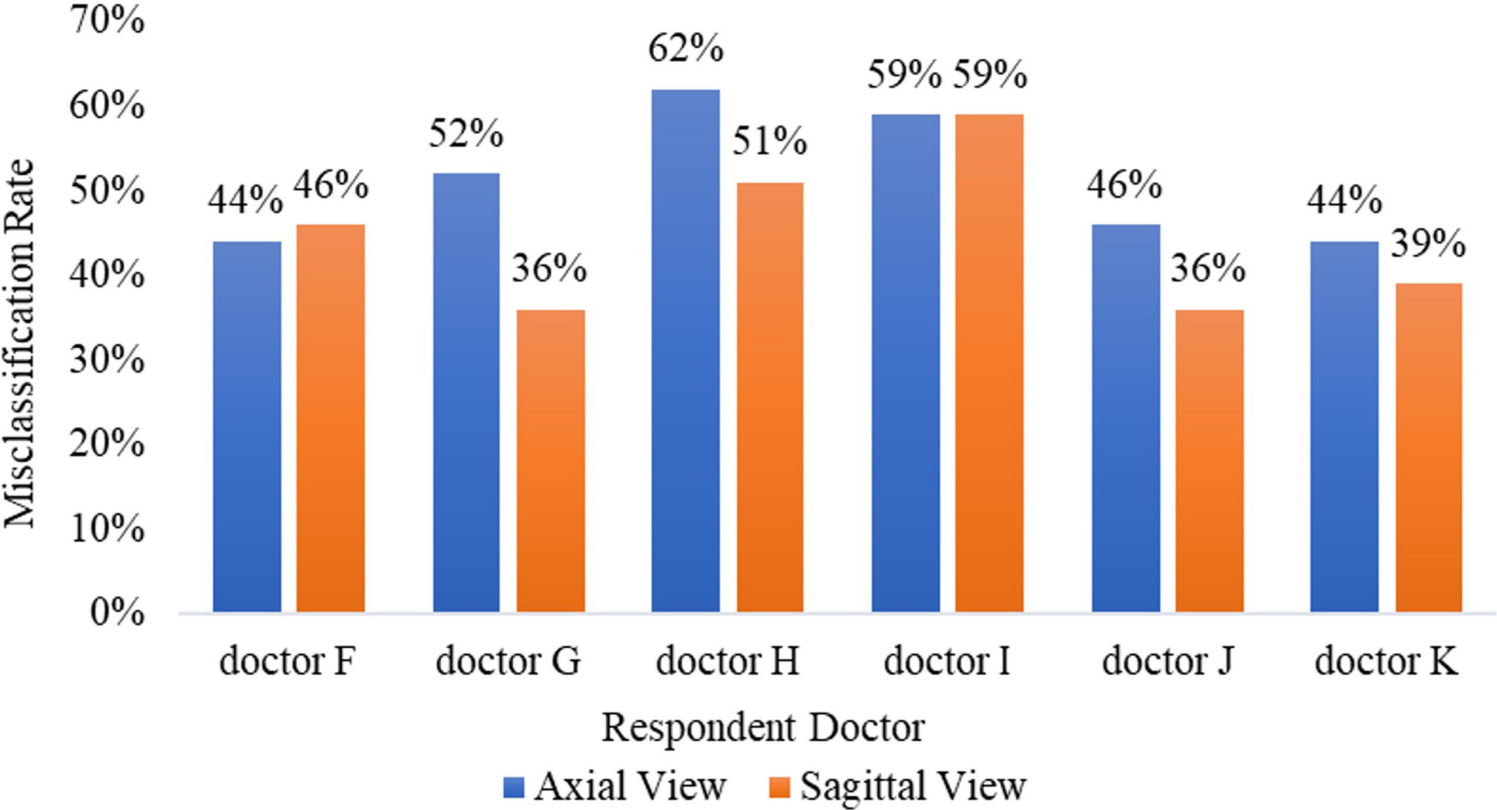
The misclassification rates of all six respondents in axial and sagittal views.

**Figure 9:**
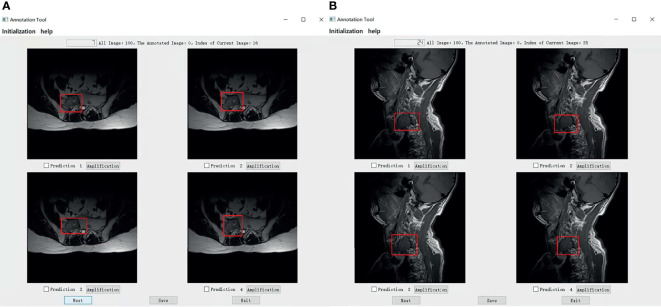
**(A)** Shows that all doctors F-K have selected prediction 2 which is predicted by the model CNN1 instead of prediction 4 annotated by one of the doctors A-E in axial. **(B)** Shows that four of all doctors F-K have selected the predictions from the models instead of the prediction 3 annotated by one of the doctor A-E in the signal. Among them, four of all doctors F-K chose prediction 4 from the model CNN3, they were doctors F, H, I, and J And doctor G chose prediction 1 predicted by CNN1, and doctor K chose the prediction 2 predicted by CNN2.

**Figure 10:**
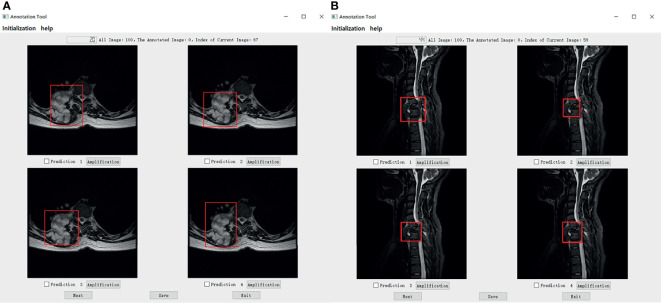
**(A)** Shows that all doctors F-K have correctly selected the prediction 4 annotated by doctors A-E in the axial. And **(B)** shows that all doctors F-K have correctly selected the prediction 1 annotated by doctors A-E in sagittal.

The mean Average Precision (mAP) results of CNN1, CNN2, and CNN3 were 79.1%, 79.8%, and 80.6% respectively in the axial view, and 84.5%, 85.2%, and 86.1% in the sagittal view, respectively when IoU was over 0.3. These three models were used for Turing testing. The Turing test contained 100 choice questions in the axial view and another 100 choice questions in the sagittal view. **Figure 8** shows the overall percentage of annotation images incorrectly identified by each respondent when asked the following: “Which one was drawn by a human?” in axial and sagittal views. The misclassification rates for the respondents were 44% (F), 52% (G), 62% (H), 59% (I), 46% (J), and 44% (K) in the axial view question, and the average misclassification rate was 51.2% (95% CI: 45.7–57.5%). Among the results of doctors who wrongly selected the prediction of the DL model but did not correctly select the annotations of the doctors A-E, 47.6% chose the prediction by CNN3, 27.4% by CNN2, and 25.0% by CNN1 in the axial view question. Moreover, the misclassification rates for the respondents were 46% (F), 36% (G), 51% (H), 59% (I), 36% (J), and 39% (K) in the sagittal view question, and the average misclassification rate was 44.5% (95% CI: 38.2–51.8%). Among the results of doctors who wrongly selected the prediction of the DL model but did not correctly select the annotations of the doctors A-E, 48.4% chose the prediction by CNN3, 26.2% by CNN2, and 25.4% by CNN1 in the sagittal view question. According to the results selected by doctors F-K, CNN 3 performed better and the predictions were closer to the manual annotations. Among the six respondents, the lowest misclassification was achieved by an expert radiologist with 25 years of experience. The misclassification rates of the respondents during the Turing test represented an inability to distinguish the annotation source between a human and a computer. The misclassification rates were all >30%, indicating that the DL models passed the Turing test. Therefore, the automated detection of spine tumors by our DL model was equal to that of annotation doctors in our hospital. The complete raw results from the Turing test are provided as Supplemental Material (see file “TuringTestResults”). **Figure 9** shows an MRI scan in which all doctors chose the DL prediction in both axial and sagittal views, which indicated their failure. **Figure 10** shows an MRI scan in which all doctors F-K correctly selected the annotations of doctors A-E in axial and sagittal views, respectively.

**Table 3** shows the assessment time required by each respondent for each multiple-choice question in the Turing test. In the axial view, the average time per question needed by each respondent for the Turing test was 10.72 s (F), 12.08 s (G), 15.73 s (H), 9.46 s (I), 5.69 s (J), and 9.01 s (K). For the 100 choice questions in the axial view, the mean time for each question was 10.45 (range: 5–70) s; therefore, the entire assessment took approximately 17 min 25 s per participant (range: 9 min 29 s–26 min 13 s). Moreover, in the sagittal view, the average time per question taken by each respondent for the Turing test was 9.25 s (F), 13.92 s (G), 10.04 s (H), 9.66 s (I), 7.41 s (J), and 9.02 s (K). For the 100 choice questions in the sagittal view, the mean time for each question was 9.88 (range: 4–54) s; therefore, the entire assessment took approximately 16 min 28 s per participant (range: 12 min 21 s–23 min 12 s). No correlation was observed between the time required and the level of accuracy of the assessment. All time results are provided as Supplemental Material (see file “TimingResults”).

**Table 3:** The Average Time (second) Per Question Taken by Each Respondent in the Turing test.

	Doctor F	Doctor G	Doctor H	Doctor I	Doctor J	Doctor K
**Axial view(s)**	10.72	12.08	15.73	9.46	5.69	9.01
**Sagittal view(s)**	9.25	13.92	10.04	9.66	7.41	9.02

## Discussion

Rather than assessing the performance of our DL model, this study aimed primarily to evaluate whether our DL model for the automated detection of primary spine tumors was as good as that of standard manual annotation methods using the Turing test (18, 19). Although it is doubtful whether AI will ever pass the Turing test for various complex clinical scenarios, it is easy to misunderstand the role of AI in future medical development. AI should complement rather than replace medical professionals. One of our primary aims in using the DL model was to develop a novel method of detecting primary spine tumors from MR images, which is likely to assist orthopedists to find the spine tumors efficiently and reduce the burden on them in the future. The results showed that the accuracy of our DL automatic detection was comparable to that of annotation doctors in radiology or orthopedics. Despite some reports on the applications of AI systems for the spine (20–28), especially on MRI (29) and tumor (30), few studies used the Turing test to evaluate the automatic detection of primary spine tumors in MR images based on DL.

Regardless of symptoms and physical observations, AI facilitates the diagnosis of spine tumors over humans (31). Bluemke et al. (32) reviewed AI radiology research to make a brief guide for authors, reviewers, and interrogators. Wang et al. (33) made a multi-resolution approach for spine metastasis detection using deep Siamese neural networks. Liu et al. (34) compared radiomics with machine learning in the prediction of high-risk cytogenetic status in multiple myeloma based on MRI. The performance of our proposed automatic detection model is not only comparable to that of actual radiologists or orthopedists but also helps to minimize the possibility of overlooking tumors. Massaad et al. (35) used machine learning algorithms to assess the performance of the metastatic spine tumor frailty index. Furthermore, the application of this model can reduce the delay in diagnosing spine tumors because it responds significantly more quickly than humans. Additionally, due to time constraints, radiologists or orthopedists could not evaluate all MR images on their own; sometimes other surgeons or physicians must assess MR spine images. Fortunately, the detection rate of this system is comparable to that of annotation doctors, and the possibility of missing tumors becomes significantly less. Consequently, patients with primary spine tumors can be referred to spine tumor surgeons earlier and more safely.

Although the MR images used in this study corresponded to various spine tumor types, the object detection model achieved high accuracy. However, there are always exceptions in clinical settings. For instance, sometimes, it is difficult to identify spine tumors because of signal intensity, location, configuration, or tumor shape. Therefore, the differentiation of spine tumors in neuroimaging is not always reliable. Nevertheless, the use of MRI has facilitated the diagnosis of spine tumors. Another drawback is that if the patient is allergic to contrast agents and/or experiences renal insufficiency, an enhanced MRI scan cannot be performed. In this case, if our proposed system is used to detect spine tumors, we can determine whether other imaging modalities, such as positron emission tomography-computed tomography, should be performed. If MRI cannot be performed owing to renal dysfunction, the proposed system allows for MRI to be performed as minimally as possible.

Some individuals believe that passing the Turing test suggests that human-level intelligence can be achieved by machines. However, achieving human-level AI is still far from reality (36, 37). This study, compared to other Turing test studies to date, is one of a few to include a large number of patients with primary spine tumors and a large set of marked spine tumor MR images. The human respondents in this study had only a fair level of agreement with one another, averaging approximately 51.17% accuracy for selecting the human annotation. In a prior report from Scheuer et al. (38), the skilled human interrogators in their study had a higher sensitivity (45%) for electroencephalography spike events marked by three neurologists. However, the longer the Turing test, the bigger the challenge for a machine to satisfactorily pretend to be a human. In our test of 100 choice questions in the axial or sagittal view, which took approximately 17 min, it would be extremely difficult for a machine to mislead a clinical respondent. Additionally, one of the major challenges in clinical studies dealing with bounding box lesion annotation is to define a “gold standard.” Gooding et al. (13) made an evaluation of auto contouring in clinical practice using the Turing test, and Sathish et al. (18) compared lung segmentation and nodule detection between convolutional neural network and humans using the Turing test. Using the choice monitor, the respondents assumed the human’s label as the golden standard; hence, they tried to judge the best labels as objectively as possible. This study has demonstrated that with training, the DL model can improve its ability at tumor annotation and mislead the respondents’ judgments. In several studies, DL technology has been shown to have a reasonable ability to discriminate between abnormal construct and normal construct in the spine.

Despite a design to limit selection and respondent biases, this study has some limitations. First, the spine tumor MR images were all obtained from a single center, drawn from a cohort of documented patients, and the number of MR images utilized in this study was significantly limited. Hence, it is necessary to improve the accuracy of our system by incorporating multi-center MRI data. Despite the limited number of images, we were able to amplify the training datasets by applying random transformations (e.g., flipping and scaling) to the images. This technique has proven valuable for DL with small datasets. Another limitation was that the proposed system only analyzed and detected the location and approximate outline of spine tumors. Other relevant characteristics, such as whether a spine tumor was benign or malignant, were not recognized in our DL model. Therefore, further research of methods to identify other spine tumor characteristics is necessary. Furthermore, only axial and sagittal images were obtained in our study; hence, the addition of coronal images would improve the model’s performance. In addition, to help doctors with image annotation and follow-up, we converted the DICOM into an easy-to-read JPEG. The average misclassification rate of doctors in our current Turing test was over 35%. Despite these limitations, we believe that in the future, our system, with its high accuracy and comparable performance to clinical experts, could be applied to different settings and conditions.

## Conclusion

In conclusion, this study proposed an AI primary spine tumor detection system that passed the Turing test; respondents were unable to distinguish between our DL model and annotation doctors. The present results show that our DL model may be an efficient tool to assist radiologists or orthopedists in primary spine tumors detection, increasing efficiency and sparing time. In the future, larger multi-center datasets are necessary to increase the accuracy of our system and validate our model.

## Data Availability Statement

The original contributions presented in the study are included in the article/**Supplementary Material**. Further inquiries can be directed to the corresponding authors.

## Ethics Statement

This study was approved by the Medical Science Research Ethics Committee, and it waived the need for informed consent.

## Author Contributions

HO, FM, and JL, conceptualization, investigation, registration, data curation, visualization, formal analysis, and writing - original draft. XS, investigation, registration, data curation, formal analysis, and writing - original draft. YL, YY, NL, ST, and MY, conceptualization, investigation, methodology, and manual annotation. XL, conceptualization, methodology, project administration, resources, supervision, and validation. HY, SJ, and LJ, conceptualization, funding acquisition, project administration, resources, supervision, validation, writing - review and editing. All authors contributed to the article and approved the submitted version.

## Funding

This study was funded by the Beijing Municipal Natural Science Foundation (Z190020), Capital’s Funds for Health Improvement and Research (2020-4-40916), Clinical Medicine Plus X - Young Scholars Project, Peking University, the Fundamental Research Funds for the Central Universities (PKU2021LCXQ005), National Natural Science Foundation of China (82102638, 82171927, 81971578), and Peking University Third Hospital Clinical Key Project (BYSYZD2021040, BYSY2018003, BYSYZD2019005).

## Conflict of Interest

The authors declare that the research was conducted in the absence of any commercial or financial relationships that could be construed as a potential conflict of interest.

## Publisher’s Note

All claims expressed in this article are solely those of the authors and do not necessarily represent those of their affiliated organizations, or those of the publisher, the editors and the reviewers. Any product that may be evaluated in this article, or claim that may be made by its manufacturer, is not guaranteed or endorsed by the publisher.
