# Supplementary material for: Evaluation of Deep Learning-Based Automated Detection of Primary Spine Tumors on MRI Using the Turing Test
Source: Front Oncol. 2022 Mar 11;12:814667. doi: 10.3389/fonc.2022.814667 (PMC8962659; doi:10.3389/fonc.2022.814667)
Supplement: Supplementary file 1 [file Table_1.docx]

Supplementary Material

# TimingResults

| Doctor | Doctor F | | Doctor G | | Doctor H | | Doctor I | | Doctor J | | Doctor K | |
| --- | --- | --- | --- | --- | --- | --- | --- | --- | --- | --- | --- | --- |
| View | Axial | Sagittal | Axial | Sagittal | Axial | Sagittal | Axial | Sagittal | Axial | Sagittal | Axial | Sagittal |
| Time (s) | 42 | 14 | 4 | 28 | 7 | 10 | 11 | 10 | 4 | 5 | 10 | 14 |
|  | 7 | 12 | 6 | 16 | 22 | 10 | 8 | 4 | 8 | 5 | 9 | 12 |
|  | 5 | 54 | 8 | 12 | 53 | 7 | 4 | 10 | 8 | 5 | 8 | 54 |
|  | 7 | 18 | 16 | 6 | 10 | 13 | 22 | 7 | 10 | 6 | 9 | 18 |
|  | 6 | 6 | 16 | 16 | 13 | 8 | 20 | 16 | 3 | 7 | 11 | 6 |
|  | 6 | 9 | 6 | 6 | 8 | 12 | 12 | 7 | 7 | 13 | 5 | 9 |
|  | 21 | 14 | 10 | 22 | 9 | 12 | 16 | 12 | 5 | 11 | 15 | 14 |
|  | 5 | 13 | 6 | 22 | 15 | 7 | 5 | 10 | 5 | 6 | 9 | 13 |
|  | 11 | 6 | 26 | 36 | 17 | 17 | 10 | 17 | 5 | 6 | 9 | 6 |
|  | 4 | 9 | 6 | 8 | 13 | 5 | 10 | 8 | 5 | 6 | 9 | 9 |
|  | 6 | 5 | 32 | 12 | 16 | 19 | 38 | 16 | 6 | 8 | 7 | 5 |
|  | 4 | 11 | 6 | 20 | 15 | 17 | 7 | 21 | 6 | 6 | 6 | 11 |
|  | 5 | 8 | 14 | 12 | 17 | 13 | 8 | 10 | 6 | 4 | 6 | 8 |
|  | 7 | 11 | 36 | 6 | 14 | 8 | 11 | 14 | 6 | 10 | 6 | 11 |
|  | 10 | 7 | 8 | 14 | 13 | 6 | 10 | 5 | 3 | 10 | 7 | 7 |
|  | 8 | 9 | 26 | 20 | 9 | 5 | 12 | 13 | 7 | 14 | 8 | 6 |
|  | 10 | 5 | 4 | 12 | 13 | 12 | 11 | 10 | 11 | 8 | 9 | 5 |
|  | 33 | 13 | 8 | 10 | 9 | 11 | 5 | 6 | 6 | 15 | 10 | 13 |
|  | 19 | 6 | 6 | 10 | 8 | 8 | 6 | 17 | 7 | 6 | 10 | 6 |
|  | 9 | 17 | 10 | 8 | 9 | 10 | 17 | 8 | 5 | 9 | 7 | 17 |
|  | 6 | 9 | 44 | 12 | 20 | 11 | 8 | 15 | 4 | 7 | 6 | 9 |
|  | 15 | 14 | 4 | 12 | 16 | 12 | 7 | 6 | 6 | 5 | 7 | 14 |
|  | 7 | 8 | 10 | 22 | 11 | 15 | 7 | 19 | 4 | 7 | 7 | 8 |
|  | 15 | 28 | 6 | 44 | 21 | 8 | 5 | 5 | 8 | 8 | 8 | 28 |
|  | 8 | 6 | 4 | 14 | 15 | 7 | 7 | 5 | 5 | 9 | 6 | 6 |
|  | 5 | 8 | 8 | 14 | 21 | 19 | 12 | 6 | 4 | 6 | 7 | 8 |
|  | 10 | 6 | 26 | 6 | 15 | 11 | 6 | 32 | 5 | 7 | 7 | 6 |
|  | 8 | 5 | 14 | 14 | 9 | 7 | 5 | 9 | 5 | 4 | 5 | 5 |
|  | 8 | 7 | 26 | 20 | 7 | 9 | 4 | 8 | 4 | 7 | 7 | 8 |
|  | 6 | 4 | 12 | 10 | 13 | 7 | 5 | 4 | 5 | 7 | 20 | 4 |
|  | 6 | 11 | 4 | 14 | 13 | 11 | 6 | 8 | 5 | 7 | 8 | 11 |
|  | 5 | 9 | 8 | 6 | 20 | 9 | 7 | 6 | 6 | 9 | 7 | 9 |
|  | 7 | 13 | 16 | 14 | 9 | 10 | 8 | 8 | 7 | 7 | 9 | 13 |
|  | 5 | 18 | 12 | 8 | 18 | 6 | 5 | 18 | 5 | 37 | 15 | 18 |
|  | 11 | 6 | 16 | 16 | 6 | 6 | 8 | 7 | 5 | 14 | 9 | 6 |
|  | 13 | 6 | 6 | 4 | 13 | 5 | 7 | 17 | 6 | 9 | 8 | 6 |
|  | 11 | 7 | 6 | 28 | 11 | 8 | 17 | 6 | 8 | 5 | 9 | 7 |
|  | 6 | 6 | 10 | 14 | 7 | 7 | 12 | 10 | 8 | 6 | 10 | 6 |
|  | 8 | 12 | 8 | 20 | 9 | 7 | 11 | 6 | 5 | 5 | 8 | 12 |
|  | 5 | 8 | 6 | 16 | 9 | 6 | 11 | 7 | 6 | 5 | 16 | 8 |
|  | 6 | 25 | 4 | 4 | 5 | 12 | 8 | 14 | 4 | 7 | 7 | 25 |
|  | 7 | 12 | 4 | 12 | 11 | 13 | 5 | 14 | 7 | 6 | 12 | 12 |
|  | 3 | 4 | 4 | 4 | 9 | 6 | 8 | 5 | 4 | 7 | 15 | 4 |
|  | 5 | 5 | 8 | 16 | 23 | 10 | 17 | 19 | 5 | 8 | 7 | 5 |
|  | 9 | 17 | 18 | 18 | 70 | 8 | 5 | 6 | 11 | 11 | 23 | 17 |
|  | 6 | 4 | 8 | 14 | 27 | 7 | 12 | 11 | 6 | 4 | 8 | 4 |
|  | 8 | 5 | 8 | 16 | 42 | 9 | 5 | 4 | 5 | 5 | 7 | 5 |
|  | 7 | 7 | 10 | 6 | 20 | 6 | 11 | 10 | 7 | 6 | 7 | 7 |
|  | 15 | 6 | 8 | 10 | 26 | 7 | 6 | 12 | 9 | 9 | 9 | 6 |
|  | 9 | 4 | 2 | 18 | 19 | 8 | 5 | 4 | 4 | 14 | 10 | 4 |
|  | 8 | 8 | 6 | 6 | 20 | 6 | 6 | 11 | 4 | 5 | 9 | 8 |
|  | 32 | 9 | 28 | 8 | 10 | 6 | 8 | 12 | 4 | 4 | 9 | 9 |
|  | 11 | 8 | 14 | 8 | 8 | 5 | 8 | 8 | 4 | 4 | 9 | 8 |
|  | 14 | 5 | 4 | 14 | 8 | 8 | 7 | 8 | 7 | 6 | 9 | 5 |
|  | 8 | 5 | 10 | 10 | 13 | 9 | 6 | 9 | 4 | 9 | 6 | 5 |
|  | 11 | 3 | 28 | 6 | 8 | 5 | 8 | 13 | 5 | 4 | 11 | 3 |
|  | 17 | 11 | 4 | 8 | 14 | 6 | 46 | 6 | 6 | 6 | 8 | 11 |
|  | 12 | 4 | 38 | 8 | 9 | 13 | 5 | 4 | 5 | 33 | 10 | 4 |
|  | 60 | 6 | 30 | 18 | 12 | 8 | 7 | 39 | 7 | 9 | 6 | 6 |
|  | 5 | 6 | 16 | 38 | 18 | 6 | 7 | 10 | 4 | 6 | 10 | 6 |
|  | 6 | 11 | 10 | 14 | 14 | 12 | 8 | 8 | 5 | 7 | 7 | 11 |
|  | 4 | 4 | 16 | 18 | 13 | 13 | 5 | 6 | 4 | 6 | 6 | 4 |
|  | 16 | 8 | 14 | 8 | 11 | 4 | 5 | 15 | 6 | 6 | 10 | 8 |
|  | 9 | 5 | 12 | 12 | 12 | 9 | 8 | 7 | 7 | 6 | 12 | 5 |
|  | 8 | 4 | 14 | 16 | 10 | 8 | 6 | 9 | 5 | 8 | 9 | 4 |
|  | 14 | 5 | 10 | 16 | 11 | 15 | 8 | 5 | 6 | 5 | 6 | 5 |
|  | 15 | 8 | 14 | 14 | 12 | 24 | 38 | 9 | 4 | 6 | 15 | 8 |
|  | 5 | 5 | 4 | 12 | 18 | 7 | 12 | 11 | 5 | 5 | 5 | 5 |
|  | 6 | 7 | 6 | 8 | 13 | 8 | 14 | 6 | 4 | 7 | 15 | 7 |
|  | 12 | 9 | 20 | 6 | 7 | 8 | 9 | 6 | 5 | 5 | 11 | 8 |
|  | 10 | 7 | 6 | 7 | 11 | 16 | 14 | 9 | 5 | 5 | 7 | 7 |
|  | 3 | 5 | 56 | 18 | 6 | 7 | 7 | 11 | 4 | 3 | 7 | 5 |
|  | 7 | 4 | 14 | 16 | 9 | 13 | 7 | 6 | 5 | 10 | 6 | 4 |
|  | 17 | 5 | 4 | 12 | 12 | 6 | 13 | 10 | 6 | 5 | 17 | 5 |
|  | 5 | 28 | 6 | 18 | 10 | 8 | 10 | 8 | 7 | 6 | 8 | 8 |
|  | 10 | 4 | 58 | 12 | 8 | 6 | 8 | 17 | 4 | 5 | 9 | 4 |
|  | 5 | 4 | 6 | 8 | 48 | 7 | 13 | 8 | 7 | 8 | 15 | 4 |
|  | 6 | 3 | 2 | 48 | 9 | 28 | 7 | 9 | 4 | 5 | 6 | 3 |
|  | 6 | 20 | 4 | 20 | 6 | 10 | 6 | 6 | 5 | 6 | 11 | 20 |
|  | 20 | 8 | 22 | 20 | 44 | 10 | 11 | 9 | 4 | 10 | 7 | 8 |
|  | 8 | 4 | 8 | 18 | 20 | 13 | 7 | 7 | 5 | 5 | 7 | 4 |
|  | 16 | 6 | 2 | 8 | 12 | 12 | 4 | 6 | 8 | 5 | 6 | 6 |
|  | 10 | 8 | 10 | 12 | 8 | 20 | 7 | 7 | 6 | 6 | 8 | 8 |
|  | 7 | 4 | 4 | 20 | 57 | 5 | 6 | 9 | 7 | 6 | 7 | 4 |
|  | 5 | 10 | 4 | 6 | 15 | 8 | 6 | 5 | 6 | 8 | 12 | 10 |
|  | 8 | 4 | 8 | 10 | 8 | 15 | 6 | 8 | 7 | 5 | 9 | 4 |
|  | 8 | 11 | 20 | 10 | 13 | 16 | 6 | 8 | 7 | 11 | 8 | 11 |
|  | 25 | 3 | 6 | 9 | 43 | 17 | 6 | 7 | 5 | 5 | 7 | 3 |
|  | 6 | 7 | 2 | 14 | 14 | 5 | 6 | 8 | 4 | 6 | 7 | 7 |
|  | 10 | 9 | 10 | 9 | 6 | 12 | 8 | 9 | 5 | 7 | 9 | 11 |
|  | 10 | 5 | 8 | 8 | 17 | 8 | 11 | 11 | 4 | 6 | 15 | 5 |
|  | 29 | 17 | 8 | 8 | 8 | 9 | 6 | 6 | 7 | 5 | 11 | 15 |
|  | 5 | 8 | 4 | 10 | 7 | 10 | 7 | 10 | 5 | 7 | 12 | 8 |
|  | 5 | 12 | 22 | 10 | 48 | 11 | 4 | 6 | 5 | 6 | 10 | 12 |
|  | 46 | 12 | 4 | 15 | 10 | 12 | 6 | 5 | 7 | 6 | 8 | 12 |
|  | 8 | 6 | 8 | 30 | 12 | 25 | 8 | 6 | 4 | 6 | 6 | 6 |
|  | 6 | 5 | 12 | 12 | 21 | 7 | 6 | 4 | 12 | 4 | 8 | 5 |
|  | 7 | 13 | 8 | 12 | 19 | 14 | 6 | 5 | 7 | 5 | 6 | 13 |
|  | 7 | 23 | 8 | 14 | 15 | 6 | 10 | 6 | 5 | 8 | 6 | 23 |
|  | 19 | 12 | 2 | 8 | 11 | 11 | 20 | 16 | 5 | 6 | 9 | 12 |
| Average time (s) | 10.72 | 9.25 | 12.08 | 13.92 | 15.73 | 10.04 | 9.46 | 9.66 | 5.69 | 7.41 | 9.01 | 9.02 |

# Turing Test Results

| **Note: If the result is 1, it means that the doctor answered the question correctly, and if the result is 0, it means that the doctor answered the question incorrectly.** | | | | | | | | | | | | | | | | | | | | | | | |
| --- | --- | --- | --- | --- | --- | --- | --- | --- | --- | --- | --- | --- | --- | --- | --- | --- | --- | --- | --- | --- | --- | --- | --- |
| Doctor F | | | | Doctor G | | | | Doctor H | | | | Doctor I | | | | Doctor J | | | | Doctor K | | | |
| Axial | | Sagittal | | Axial | | Sagittal | | Axial | | Sagittal | | Axial | | Sagittal | | Axial | | Sagittal | | Axial | | Sagittal | |
| Question | Result | Question | Result | Question | Result | Question | Result | Question | Result | Question | Result | Question | Result | Question | Result | Question | Result | Question | Result | Question | Result | Question | Result |
| 0 | 1 | 0 | 0 | 0 | 1 | 0 | 1 | 0 | 0 | 0 | 1 | 0 | 1 | 0 | 1 | 0 | 1 | 0 | 0 | 0 | 1 | 0 | 1 |
| 1 | 0 | 1 | 0 | 1 | 1 | 1 | 1 | 1 | 0 | 1 | 0 | 1 | 0 | 1 | 0 | 1 | 0 | 1 | 1 | 1 | 0 | 1 | 1 |
| 2 | 0 | 2 | 1 | 2 | 0 | 2 | 1 | 2 | 1 | 2 | 1 | 2 | 0 | 2 | 0 | 2 | 0 | 2 | 1 | 2 | 1 | 2 | 1 |
| 3 | 1 | 3 | 1 | 3 | 1 | 3 | 1 | 3 | 0 | 3 | 1 | 3 | 1 | 3 | 1 | 3 | 0 | 3 | 0 | 3 | 1 | 3 | 0 |
| 4 | 1 | 4 | 1 | 4 | 1 | 4 | 0 | 4 | 1 | 4 | 1 | 4 | 0 | 4 | 0 | 4 | 1 | 4 | 1 | 4 | 1 | 4 | 1 |
| 5 | 0 | 5 | 1 | 5 | 0 | 5 | 1 | 5 | 0 | 5 | 1 | 5 | 0 | 5 | 0 | 5 | 1 | 5 | 1 | 5 | 1 | 5 | 1 |
| 6 | 0 | 6 | 0 | 6 | 0 | 6 | 0 | 6 | 0 | 6 | 0 | 6 | 0 | 6 | 0 | 6 | 0 | 6 | 0 | 6 | 0 | 6 | 0 |
| 7 | 0 | 7 | 1 | 7 | 0 | 7 | 1 | 7 | 0 | 7 | 1 | 7 | 0 | 7 | 0 | 7 | 0 | 7 | 1 | 7 | 0 | 7 | 1 |
| 8 | 1 | 8 | 0 | 8 | 0 | 8 | 0 | 8 | 0 | 8 | 0 | 8 | 1 | 8 | 1 | 8 | 1 | 8 | 1 | 8 | 1 | 8 | 0 |
| 9 | 1 | 9 | 0 | 9 | 1 | 9 | 1 | 9 | 1 | 9 | 0 | 9 | 0 | 9 | 0 | 9 | 1 | 9 | 1 | 9 | 1 | 9 | 0 |
| 10 | 1 | 10 | 1 | 10 | 0 | 10 | 1 | 10 | 1 | 10 | 0 | 10 | 1 | 10 | 1 | 10 | 1 | 10 | 1 | 10 | 1 | 10 | 1 |
| 11 | 0 | 11 | 1 | 11 | 0 | 11 | 1 | 11 | 0 | 11 | 1 | 11 | 0 | 11 | 0 | 11 | 0 | 11 | 1 | 11 | 0 | 11 | 1 |
| 12 | 1 | 12 | 1 | 12 | 1 | 12 | 0 | 12 | 1 | 12 | 1 | 12 | 1 | 12 | 1 | 12 | 1 | 12 | 1 | 12 | 0 | 12 | 0 |
| 13 | 1 | 13 | 1 | 13 | 1 | 13 | 1 | 13 | 0 | 13 | 0 | 13 | 1 | 13 | 1 | 13 | 0 | 13 | 0 | 13 | 1 | 13 | 0 |
| 14 | 1 | 14 | 1 | 14 | 0 | 14 | 0 | 14 | 1 | 14 | 0 | 14 | 0 | 14 | 0 | 14 | 1 | 14 | 1 | 14 | 1 | 14 | 0 |
| 15 | 0 | 15 | 1 | 15 | 0 | 15 | 1 | 15 | 0 | 15 | 1 | 15 | 0 | 15 | 0 | 15 | 0 | 15 | 1 | 15 | 0 | 15 | 1 |
| 16 | 0 | 16 | 0 | 16 | 1 | 16 | 1 | 16 | 0 | 16 | 1 | 16 | 0 | 16 | 0 | 16 | 0 | 16 | 1 | 16 | 0 | 16 | 1 |
| 17 | 1 | 17 | 1 | 17 | 1 | 17 | 1 | 17 | 1 | 17 | 1 | 17 | 1 | 17 | 1 | 17 | 1 | 17 | 1 | 17 | 1 | 17 | 1 |
| 18 | 1 | 18 | 0 | 18 | 1 | 18 | 0 | 18 | 1 | 18 | 1 | 18 | 1 | 18 | 1 | 18 | 1 | 18 | 0 | 18 | 1 | 18 | 1 |
| 19 | 0 | 19 | 1 | 19 | 1 | 19 | 1 | 19 | 0 | 19 | 1 | 19 | 0 | 19 | 0 | 19 | 1 | 19 | 1 | 19 | 1 | 19 | 1 |
| 20 | 1 | 20 | 0 | 20 | 1 | 20 | 0 | 20 | 1 | 20 | 0 | 20 | 0 | 20 | 0 | 20 | 1 | 20 | 0 | 20 | 0 | 20 | 0 |
| 21 | 1 | 21 | 0 | 21 | 1 | 21 | 1 | 21 | 1 | 21 | 1 | 21 | 1 | 21 | 1 | 21 | 0 | 21 | 1 | 21 | 0 | 21 | 1 |
| 22 | 1 | 22 | 0 | 22 | 0 | 22 | 1 | 22 | 0 | 22 | 0 | 22 | 1 | 22 | 1 | 22 | 0 | 22 | 0 | 22 | 0 | 22 | 0 |
| 23 | 1 | 23 | 1 | 23 | 0 | 23 | 1 | 23 | 1 | 23 | 0 | 23 | 1 | 23 | 1 | 23 | 1 | 23 | 1 | 23 | 0 | 23 | 1 |
| 24 | 1 | 24 | 1 | 24 | 0 | 24 | 1 | 24 | 1 | 24 | 0 | 24 | 1 | 24 | 1 | 24 | 0 | 24 | 1 | 24 | 1 | 24 | 0 |
| 25 | 0 | 25 | 0 | 25 | 0 | 25 | 1 | 25 | 0 | 25 | 0 | 25 | 0 | 25 | 0 | 25 | 1 | 25 | 1 | 25 | 1 | 25 | 1 |
| 26 | 0 | 26 | 0 | 26 | 0 | 26 | 0 | 26 | 0 | 26 | 1 | 26 | 0 | 26 | 0 | 26 | 0 | 26 | 0 | 26 | 0 | 26 | 1 |
| 27 | 0 | 27 | 1 | 27 | 0 | 27 | 1 | 27 | 0 | 27 | 1 | 27 | 0 | 27 | 0 | 27 | 0 | 27 | 1 | 27 | 0 | 27 | 1 |
| 28 | 0 | 28 | 0 | 28 | 1 | 28 | 1 | 28 | 0 | 28 | 1 | 28 | 0 | 28 | 0 | 28 | 1 | 28 | 1 | 28 | 0 | 28 | 0 |
| 29 | 0 | 29 | 1 | 29 | 0 | 29 | 1 | 29 | 1 | 29 | 0 | 29 | 0 | 29 | 0 | 29 | 1 | 29 | 1 | 29 | 0 | 29 | 1 |
| 30 | 1 | 30 | 0 | 30 | 0 | 30 | 1 | 30 | 0 | 30 | 0 | 30 | 0 | 30 | 0 | 30 | 0 | 30 | 1 | 30 | 0 | 30 | 1 |
| 31 | 1 | 31 | 1 | 31 | 1 | 31 | 1 | 31 | 0 | 31 | 1 | 31 | 0 | 31 | 0 | 31 | 1 | 31 | 1 | 31 | 0 | 31 | 1 |
| 32 | 1 | 32 | 1 | 32 | 1 | 32 | 1 | 32 | 1 | 32 | 1 | 32 | 0 | 32 | 0 | 32 | 1 | 32 | 1 | 32 | 1 | 32 | 1 |
| 33 | 1 | 33 | 1 | 33 | 0 | 33 | 1 | 33 | 1 | 33 | 1 | 33 | 1 | 33 | 1 | 33 | 0 | 33 | 1 | 33 | 1 | 33 | 1 |
| 34 | 0 | 34 | 0 | 34 | 1 | 34 | 0 | 34 | 0 | 34 | 0 | 34 | 1 | 34 | 1 | 34 | 0 | 34 | 0 | 34 | 1 | 34 | 0 |
| 35 | 0 | 35 | 0 | 35 | 0 | 35 | 0 | 35 | 0 | 35 | 1 | 35 | 0 | 35 | 0 | 35 | 1 | 35 | 0 | 35 | 1 | 35 | 0 |
| 36 | 1 | 36 | 1 | 36 | 1 | 36 | 0 | 36 | 0 | 36 | 0 | 36 | 0 | 36 | 0 | 36 | 1 | 36 | 1 | 36 | 1 | 36 | 1 |
| 37 | 0 | 37 | 1 | 37 | 0 | 37 | 1 | 37 | 0 | 37 | 0 | 37 | 1 | 37 | 1 | 37 | 0 | 37 | 1 | 37 | 0 | 37 | 1 |
| 38 | 1 | 38 | 0 | 38 | 0 | 38 | 0 | 38 | 1 | 38 | 0 | 38 | 0 | 38 | 0 | 38 | 0 | 38 | 1 | 38 | 0 | 38 | 1 |
| 39 | 0 | 39 | 0 | 39 | 0 | 39 | 0 | 39 | 0 | 39 | 0 | 39 | 1 | 39 | 1 | 39 | 1 | 39 | 0 | 39 | 1 | 39 | 0 |
| 40 | 1 | 40 | 1 | 40 | 0 | 40 | 1 | 40 | 0 | 40 | 0 | 40 | 0 | 40 | 0 | 40 | 0 | 40 | 1 | 40 | 1 | 40 | 0 |
| 41 | 1 | 41 | 0 | 41 | 1 | 41 | 0 | 41 | 0 | 41 | 0 | 41 | 0 | 41 | 0 | 41 | 1 | 41 | 1 | 41 | 0 | 41 | 0 |
| 42 | 0 | 42 | 0 | 42 | 0 | 42 | 0 | 42 | 1 | 42 | 1 | 42 | 1 | 42 | 1 | 42 | 0 | 42 | 0 | 42 | 0 | 42 | 0 |
| 43 | 1 | 43 | 0 | 43 | 0 | 43 | 1 | 43 | 1 | 43 | 1 | 43 | 1 | 43 | 1 | 43 | 0 | 43 | 1 | 43 | 1 | 43 | 0 |
| 44 | 1 | 44 | 0 | 44 | 0 | 44 | 1 | 44 | 0 | 44 | 0 | 44 | 0 | 44 | 0 | 44 | 1 | 44 | 1 | 44 | 0 | 44 | 1 |
| 45 | 0 | 45 | 1 | 45 | 0 | 45 | 1 | 45 | 0 | 45 | 1 | 45 | 0 | 45 | 0 | 45 | 1 | 45 | 1 | 45 | 0 | 45 | 1 |
| 46 | 1 | 46 | 0 | 46 | 1 | 46 | 0 | 46 | 1 | 46 | 0 | 46 | 1 | 46 | 1 | 46 | 1 | 46 | 0 | 46 | 1 | 46 | 1 |
| 47 | 1 | 47 | 0 | 47 | 0 | 47 | 1 | 47 | 0 | 47 | 0 | 47 | 1 | 47 | 1 | 47 | 1 | 47 | 1 | 47 | 1 | 47 | 1 |
| 48 | 0 | 48 | 0 | 48 | 0 | 48 | 1 | 48 | 0 | 48 | 0 | 48 | 0 | 48 | 0 | 48 | 0 | 48 | 0 | 48 | 0 | 48 | 1 |
| 49 | 0 | 49 | 1 | 49 | 1 | 49 | 1 | 49 | 0 | 49 | 1 | 49 | 0 | 49 | 0 | 49 | 0 | 49 | 1 | 49 | 0 | 49 | 1 |
| 50 | 1 | 50 | 1 | 50 | 0 | 50 | 0 | 50 | 1 | 50 | 0 | 50 | 1 | 50 | 1 | 50 | 1 | 50 | 1 | 50 | 1 | 50 | 0 |
| 51 | 0 | 51 | 1 | 51 | 1 | 51 | 1 | 51 | 0 | 51 | 0 | 51 | 0 | 51 | 0 | 51 | 1 | 51 | 1 | 51 | 0 | 51 | 1 |
| 52 | 0 | 52 | 1 | 52 | 1 | 52 | 0 | 52 | 0 | 52 | 0 | 52 | 0 | 52 | 0 | 52 | 0 | 52 | 1 | 52 | 0 | 52 | 0 |
| 53 | 0 | 53 | 0 | 53 | 0 | 53 | 1 | 53 | 1 | 53 | 0 | 53 | 1 | 53 | 1 | 53 | 0 | 53 | 0 | 53 | 0 | 53 | 1 |
| 54 | 1 | 54 | 1 | 54 | 1 | 54 | 1 | 54 | 1 | 54 | 1 | 54 | 1 | 54 | 1 | 54 | 1 | 54 | 1 | 54 | 1 | 54 | 1 |
| 55 | 0 | 55 | 1 | 55 | 0 | 55 | 0 | 55 | 0 | 55 | 0 | 55 | 0 | 55 | 0 | 55 | 0 | 55 | 1 | 55 | 1 | 55 | 1 |
| 56 | 0 | 56 | 0 | 56 | 0 | 56 | 0 | 56 | 1 | 56 | 0 | 56 | 0 | 56 | 0 | 56 | 1 | 56 | 0 | 56 | 0 | 56 | 0 |
| 57 | 0 | 57 | 1 | 57 | 0 | 57 | 0 | 57 | 0 | 57 | 1 | 57 | 0 | 57 | 0 | 57 | 0 | 57 | 0 | 57 | 0 | 57 | 0 |
| 58 | 1 | 58 | 1 | 58 | 1 | 58 | 1 | 58 | 0 | 58 | 1 | 58 | 0 | 58 | 0 | 58 | 0 | 58 | 1 | 58 | 1 | 58 | 1 |
| 59 | 1 | 59 | 1 | 59 | 1 | 59 | 1 | 59 | 0 | 59 | 1 | 59 | 0 | 59 | 0 | 59 | 0 | 59 | 1 | 59 | 0 | 59 | 1 |
| 60 | 1 | 60 | 0 | 60 | 1 | 60 | 1 | 60 | 0 | 60 | 0 | 60 | 1 | 60 | 1 | 60 | 1 | 60 | 0 | 60 | 1 | 60 | 1 |
| 61 | 1 | 61 | 0 | 61 | 1 | 61 | 1 | 61 | 0 | 61 | 1 | 61 | 0 | 61 | 0 | 61 | 1 | 61 | 0 | 61 | 1 | 61 | 1 |
| 62 | 1 | 62 | 0 | 62 | 0 | 62 | 0 | 62 | 0 | 62 | 0 | 62 | 1 | 62 | 1 | 62 | 1 | 62 | 0 | 62 | 0 | 62 | 0 |
| 63 | 0 | 63 | 1 | 63 | 0 | 63 | 1 | 63 | 0 | 63 | 0 | 63 | 0 | 63 | 0 | 63 | 0 | 63 | 0 | 63 | 0 | 63 | 1 |
| 64 | 0 | 64 | 0 | 64 | 0 | 64 | 0 | 64 | 1 | 64 | 0 | 64 | 0 | 64 | 0 | 64 | 1 | 64 | 1 | 64 | 1 | 64 | 1 |
| 65 | 1 | 65 | 0 | 65 | 1 | 65 | 1 | 65 | 1 | 65 | 0 | 65 | 1 | 65 | 1 | 65 | 1 | 65 | 0 | 65 | 1 | 65 | 1 |
| 66 | 0 | 66 | 1 | 66 | 0 | 66 | 0 | 66 | 0 | 66 | 0 | 66 | 0 | 66 | 0 | 66 | 1 | 66 | 1 | 66 | 1 | 66 | 1 |
| 67 | 1 | 67 | 0 | 67 | 1 | 67 | 0 | 67 | 0 | 67 | 0 | 67 | 1 | 67 | 1 | 67 | 1 | 67 | 0 | 67 | 1 | 67 | 0 |
| 68 | 1 | 68 | 1 | 68 | 1 | 68 | 1 | 68 | 1 | 68 | 1 | 68 | 0 | 68 | 0 | 68 | 1 | 68 | 1 | 68 | 0 | 68 | 0 |
| 69 | 1 | 69 | 0 | 69 | 0 | 69 | 0 | 69 | 0 | 69 | 1 | 69 | 1 | 69 | 1 | 69 | 1 | 69 | 0 | 69 | 1 | 69 | 0 |
| 70 | 1 | 70 | 1 | 70 | 1 | 70 | 1 | 70 | 0 | 70 | 1 | 70 | 0 | 70 | 0 | 70 | 0 | 70 | 0 | 70 | 1 | 70 | 1 |
| 71 | 0 | 71 | 0 | 71 | 0 | 71 | 1 | 71 | 0 | 71 | 1 | 71 | 0 | 71 | 0 | 71 | 0 | 71 | 0 | 71 | 0 | 71 | 0 |
| 72 | 1 | 72 | 1 | 72 | 1 | 72 | 1 | 72 | 0 | 72 | 1 | 72 | 1 | 72 | 1 | 72 | 1 | 72 | 1 | 72 | 0 | 72 | 1 |
| 73 | 1 | 73 | 0 | 73 | 0 | 73 | 1 | 73 | 1 | 73 | 0 | 73 | 0 | 73 | 0 | 73 | 0 | 73 | 1 | 73 | 0 | 73 | 0 |
| 74 | 1 | 74 | 1 | 74 | 1 | 74 | 1 | 74 | 1 | 74 | 1 | 74 | 1 | 74 | 1 | 74 | 1 | 74 | 1 | 74 | 1 | 74 | 1 |
| 75 | 1 | 75 | 1 | 75 | 1 | 75 | 1 | 75 | 0 | 75 | 1 | 75 | 0 | 75 | 0 | 75 | 1 | 75 | 1 | 75 | 1 | 75 | 1 |
| 76 | 1 | 76 | 1 | 76 | 1 | 76 | 1 | 76 | 1 | 76 | 1 | 76 | 1 | 76 | 1 | 76 | 1 | 76 | 1 | 76 | 1 | 76 | 0 |
| 77 | 0 | 77 | 0 | 77 | 0 | 77 | 0 | 77 | 0 | 77 | 1 | 77 | 0 | 77 | 0 | 77 | 0 | 77 | 0 | 77 | 1 | 77 | 0 |
| 78 | 1 | 78 | 1 | 78 | 1 | 78 | 1 | 78 | 1 | 78 | 0 | 78 | 1 | 78 | 1 | 78 | 1 | 78 | 0 | 78 | 1 | 78 | 0 |
| 79 | 0 | 79 | 1 | 79 | 0 | 79 | 1 | 79 | 1 | 79 | 1 | 79 | 0 | 79 | 0 | 79 | 1 | 79 | 1 | 79 | 1 | 79 | 1 |
| 80 | 1 | 80 | 0 | 80 | 1 | 80 | 1 | 80 | 0 | 80 | 0 | 80 | 1 | 80 | 1 | 80 | 0 | 80 | 1 | 80 | 1 | 80 | 1 |
| 81 | 0 | 81 | 1 | 81 | 0 | 81 | 1 | 81 | 0 | 81 | 0 | 81 | 0 | 81 | 0 | 81 | 1 | 81 | 1 | 81 | 1 | 81 | 1 |
| 82 | 0 | 82 | 0 | 82 | 0 | 82 | 1 | 82 | 0 | 82 | 0 | 82 | 0 | 82 | 0 | 82 | 0 | 82 | 0 | 82 | 1 | 82 | 1 |
| 83 | 0 | 83 | 1 | 83 | 0 | 83 | 1 | 83 | 0 | 83 | 1 | 83 | 0 | 83 | 0 | 83 | 0 | 83 | 1 | 83 | 0 | 83 | 1 |
| 84 | 1 | 84 | 0 | 84 | 0 | 84 | 0 | 84 | 0 | 84 | 0 | 84 | 0 | 84 | 0 | 84 | 0 | 84 | 1 | 84 | 1 | 84 | 0 |
| 85 | 0 | 85 | 1 | 85 | 1 | 85 | 1 | 85 | 0 | 85 | 1 | 85 | 0 | 85 | 0 | 85 | 0 | 85 | 1 | 85 | 1 | 85 | 1 |
| 86 | 1 | 86 | 1 | 86 | 1 | 86 | 1 | 86 | 1 | 86 | 0 | 86 | 1 | 86 | 1 | 86 | 0 | 86 | 1 | 86 | 0 | 86 | 1 |
| 87 | 0 | 87 | 0 | 87 | 1 | 87 | 0 | 87 | 0 | 87 | 0 | 87 | 0 | 87 | 0 | 87 | 1 | 87 | 0 | 87 | 1 | 87 | 0 |
| 88 | 1 | 88 | 1 | 88 | 1 | 88 | 1 | 88 | 1 | 88 | 1 | 88 | 1 | 88 | 1 | 88 | 1 | 88 | 1 | 88 | 1 | 88 | 1 |
| 89 | 0 | 89 | 1 | 89 | 0 | 89 | 0 | 89 | 1 | 89 | 1 | 89 | 0 | 89 | 0 | 89 | 0 | 89 | 0 | 89 | 0 | 89 | 0 |
| 90 | 1 | 90 | 0 | 90 | 1 | 90 | 0 | 90 | 0 | 90 | 0 | 90 | 1 | 90 | 1 | 90 | 1 | 90 | 0 | 90 | 1 | 90 | 0 |
| 91 | 1 | 91 | 1 | 91 | 1 | 91 | 1 | 91 | 0 | 91 | 1 | 91 | 0 | 91 | 0 | 91 | 1 | 91 | 1 | 91 | 1 | 91 | 1 |
| 92 | 1 | 92 | 1 | 92 | 0 | 92 | 1 | 92 | 0 | 92 | 1 | 92 | 0 | 92 | 0 | 92 | 1 | 92 | 1 | 92 | 1 | 92 | 1 |
| 93 | 0 | 93 | 0 | 93 | 0 | 93 | 0 | 93 | 1 | 93 | 0 | 93 | 0 | 93 | 0 | 93 | 0 | 93 | 0 | 93 | 0 | 93 | 0 |
| 94 | 0 | 94 | 0 | 94 | 0 | 94 | 0 | 94 | 0 | 94 | 1 | 94 | 1 | 94 | 1 | 94 | 0 | 94 | 0 | 94 | 0 | 94 | 0 |
| 95 | 0 | 95 | 1 | 95 | 1 | 95 | 1 | 95 | 0 | 95 | 1 | 95 | 0 | 95 | 0 | 95 | 0 | 95 | 1 | 95 | 0 | 95 | 1 |
| 96 | 1 | 96 | 0 | 96 | 1 | 96 | 0 | 96 | 1 | 96 | 0 | 96 | 1 | 96 | 1 | 96 | 1 | 96 | 0 | 96 | 1 | 96 | 0 |
| 97 | 1 | 97 | 0 | 97 | 1 | 97 | 0 | 97 | 1 | 97 | 0 | 97 | 1 | 97 | 1 | 97 | 1 | 97 | 0 | 97 | 1 | 97 | 0 |
| 98 | 0 | 98 | 1 | 98 | 0 | 98 | 1 | 98 | 0 | 98 | 1 | 98 | 1 | 98 | 1 | 98 | 0 | 98 | 1 | 98 | 0 | 98 | 1 |
| 99 | 1 | 99 | 1 | 99 | 1 | 99 | 0 | 99 | 1 | 99 | 0 | 99 | 1 | 99 | 1 | 99 | 1 | 99 | 1 | 99 | 1 | 99 | 1 |
